# Visual–Vestibular Modification of Egomotion Perception in Patients With Persistent Postural‐Perceptual Dizziness in Supine and Standing Positions

**DOI:** 10.1002/brb3.71268

**Published:** 2026-02-16

**Authors:** Skadi Gerkensmeier, Hannah Keller, Pia Herborn, Renana Storm, Christoph Helmchen, Andreas Sprenger

**Affiliations:** ^1^ Department of Neurology University Hospital Schleswig‐Holstein Lübeck Germany; ^2^ Center of Brain Behavior and Metabolism (CBBM) University of Lübeck Lübeck Germany; ^3^ Institute of Psychology II University of Lübeck Lübeck Germany

**Keywords:** body position, egomotion perception, persistent perceptual postural dizziness, vestibular, visual stimulation

## Abstract

**Background:**

Persistent postural‐perceptual dizziness (PPPD) has been classified by the Bárány Society as a chronic functional dizziness disorder with perceived unsteadiness. Symptoms typically worsen by upright posture or exposure to moving visual stimuli or complex visual patterns. The specific visual features remain poorly defined. We investigated how visual, vestibular, and combined visual–vestibular stimulation affects egomotion perception in PPPD patients, comparing responses in upright and supine positions to those of age‐matched healthy controls (HC).

**Methods:**

Participants performed a self‐rating of perceived egomotion in two sessions with two different body positions: supine and upright standing. During both sessions, participants were exposed to three types of visual stimulation designed to differ by the degree of elicited egomotion: a black‐and‐white silent movie, a flow field animation, and a rollercoaster video from the driver's perspective. Each visual stimulus was presented in combination with one of three binaural vestibular galvanic stimulation conditions (GVS): no stimulation (noGVS), fixed intensity stimulation (fixGVS: 1.3 mA), or sham (sham: 1.3 mA).

**Results:**

PPPD patients consistently reported stronger egomotion than HC, regardless of the stimulation condition. Both visual and vestibular inputs robustly influenced egomotion perception in standing and supine positions. Egomotion perception of PPPD patients in the standing, compared to the supine position, was larger during rollercoaster stimulation.

**Conclusion:**

Our findings challenge the notion of generalized visual hypersensitivity in PPPD. They rather point to a context‐dependent alteration of egomotion perception during upright stance. Differences disappear with concomitant vestibular stimulation supporting the need for vestibular rehabilitation in PPPD.

## Graphical abstract

We investigated how visual, vestibular, and combined visual–vestibular stimulation affects egomotion perception in persistent postural‐perceptual dizziness (PPPD) patients, comparing responses in upright and supine positions to those of age‐matched healthy controls (HC). PPPD patients consistently reported stronger egomotion than HC, regardless of the stimulation condition. Egomotion perception of PPPD patients in the standing, compared to the supine, position was larger during rollercoaster stimulation. Our findings point to a context‐dependent alteration of egomotion perception during upright stance.

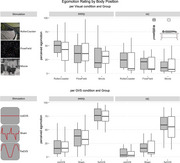

## 1 Introduction

Persistent postural‐perceptual dizziness (PPPD) is a chronic disorder characterized by perceived postural unsteadiness (Popkirov et al. 2018). Diagnostic criteria were defined by the Bárány Society in 2017 (Staab et al. 2017), with symptoms typically worsen with upright posture, active or passive motion, or exposure to moving visual stimuli or complex visual patterns. The aim of this study is to systematically investigate how these exacerbating factors influence postural sway and egomotion perception in patients with PPPD.

Abnormal visual dependence of postural control with increased reliance on visual cues may destabilize postural control and increase dizziness and anxiety in PPPD (Staab 2023). PPPD patients showed larger postural sway during static balance tests when being exposed to a Rod‐and‐Disc test that was related to clinical scores of general visual sensitivity, that is, the Visual Vertigo Analog Scale (VVAS; De Vestel et al. 2022).

Changes in visual sensitivity have been observed following vestibular disorders, for example, in acute vestibulopathy or benign paroxysmal positional vertigo (BPPV), as spatial orientation becomes more dependent on non‐vestibular sensory stimuli (Cousins et al. 2013). PPPD is often preceded by a previous transient vestibular disorder. Therefore, similar mechanisms of visual dependence have been proposed. Abnormal postural responses to visual and somatosensory stimulation have been recorded in PPPD and its precursors (De Vestel et al. 2022; McCaslin et al. 2022; Sohsten et al. 2016). This suggests that perceived postural control is altered by some visual–vestibular ambiguity in PPPD.

However, visual dependence does not necessarily imply hypersensitivity, as dizziness and anxiety in PPPD might come from abnormally elevated visual motion detection thresholds PPPD (Storm et al. 2024). Importantly, stimulus‐related perceptual aspects of postural responses of PPPD patients and its nosological precursors to experimental visual and somatosensory modifications have been less systematically assessed yet (De Vestel et al. 2022; McCaslin et al. 2022; Riccelli et al. 2017; Sohsten et al. 2016). Recordings of stimulus‐related perception are crucial as PPPD patients in upright stance perceive their postural stability much worse than objectively assessed (Murillo et al. 2023). This postural misperception may result from an increased reliance on visual input and may be missed by general clinical scores of visual sensitivity (VVAS) or ratings of the “sense of presence” at the end of the experiment (Riccelli et al. 2017).

The specific visual motion features that trigger and maintain abnormal egomotion perception in PPPD remain poorly defined. Furthermore, the influence of body posture has not yet been systematically examined, even though it is likely to be a relevant factor when comparing studies using posturography in standing position with MRI studies in the supine position. We investigated how visual, vestibular, and combined visual–vestibular stimulation affect egomotion perception in PPPD, comparing responses in upright and supine positions to those of age‐matched healthy control subjects (HC). Based on the Bárány criteria of PPPD (Staab et al. 2017), we specifically pursued three objectives: Does egomotion perception of PPPD patients differ from HC (i) by exposure to visual motion stimuli varying in their capacity of eliciting egomotion, (ii) when vestibular stimuli evoking egomotion perception are combined with visual stimuli (visual–vestibular interaction), and (iii) when supine versus standing positions are compared? These objectives take into account that standing is physiologically associated with a perception of postural sway/egomotion and that brain imaging studies rely on examinations in supine positions in which perceptional assessments have not been examined yet. We hypothesized stronger visually induced egomotion perception in PPPD patients, greater effects of vestibular over visual stimulation, and maximal responses during combined stimulation during upright stance.

## 2 Methods

Twenty‐six (eight males, 16 primary PPPDs) PPPD patients and 21 (nine males) age‐matched healthy control subjects (HC) took part in this study. Two hundred simulations of statistical power were performed using R package *simr* (Green and MacLeod 2016). For *N* = 20 for each group, the estimated power was 84.5% (78.7%–89.2% [95% CI], for details, see Supporting Information). PPPD patients were diagnosed at the Centre for Vertigo and Balance Disorders (University of Luebeck/Germany) according to the disease criteria by the Bárány Society (Staab et al. 2017). All participants underwent a neurologic and neuro‐otologic examination as part of the study (for details, see Storm et al. 2024). All of them had normal visual acuity and showed normal vestibular function on quantitative testing (head impulse test, vestibular evoked myogenic potentials, subjective visual vertical). Subjects with larger SVV deviations, possibly reflecting increased visual sensitivity, were not excluded from the study (range: 0.01–4.8°). The study protocol was approved by the local Ethics Committee of the University of Lübeck (#21‐098). Symptom severity, including visual dependency, of PPPD was assessed using the Niigata PPPD Questionnaire (NPQ; Yagi et al. 2019) and the Athens‐Lübeck Questionnaire (ALQ; Anagnostou et al. 2025).

**FIGURE 1:**
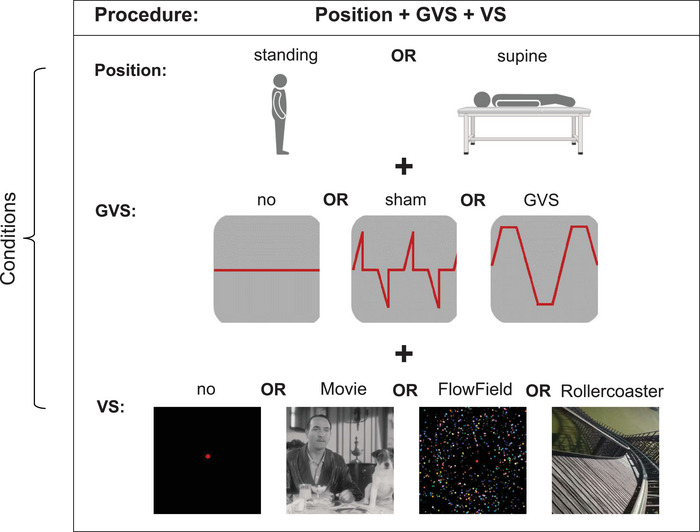
Experimental setup. Each participant was exposed to either GVS, VS, or both GVS and VS combined in standing and supine positions, producing a 2 (standing/supine) × 3 (no, sham, GVS) × 4 (no, Movie, FlowField, Rollercoaster VS) design.

Participants performed a self‐rating of perceived egomotion in two sessions with two different body positions: supine and upright standing (Figure 1). During both sessions, participants were exposed to three types of visual stimulation (VS) designed to differ by the degree of elicited egomotion: a black‐and‐white silent movie (Movie), a flow field animation (FlowField), and a roller coaster video from the driver's perspective (Rollercoaster). After each trial, participants rated their perceived egomotion on a scale from 0 (“no egomotion”) to 100 (“felt as if falling off the platform/medical couch”). Ratings were provided via joystick using a triangle‐shaped visual analog scale to facilitate intuitive rating.

Each visual stimulus was presented in combination with one of three vestibular galvanic stimulation conditions (GVS): no stimulation (noGVS), fixed intensity stimulation (fixGVS: 1.3 mA), or sham (sham: 1.3 mA). While fixGVS was applied with 100 ms linear onset and offset ramps, followed by a 300 ms stimulation plateau, the 100 ms linear onset of the *sham* stimulus was followed by 400 ms without stimulation, which does not elicit directional to and fro sway perception (Figure 1).

Both visual and GVS stimulation were designed to induce egomotion perception causing perceived unsteadiness, a key symptom of PPPD (Staab et al. 2017). However, vestibular and visual stimulation intensities are not matched, and therefore direct comparisons of their relative strength are not possible. PPPD reported that fixGVS resembled their swaying unsteadiness with some larger intensity. Importantly, the stimuli were designed to elicit a medio‐lateral direction of egomotion in both PPPD and HC as reported in previous studies (Storm et al. 2024). Prior to recording, participants were made familiar with the stimuli to minimize novelty or surprise effects. For detailed methods, see Supporting Information.

The experimental design included three within‐subject factors—POSITION (supine, standing), GVS (noGVS, fixGVS, sham), and VS (Rollercoaster, FlowField, Movie)—as well as a between‐subject factor GROUP (HC, PPPD). We first evaluated the full factorial model to characterize all potential interactions, followed by reduced models to clarify the contribution of individual factors, as stated in our hypotheses. Assumption checks indicated that the ANOVA residuals were approximately normally distributed and that group variances were comparable (QQ‐plots showed no pattern).

Furthermore, we log‐transformed the data and calculated all ANOVAs with log‐transformed and un‐transformed data. As results did not differ, we report un‐transformed data for easier interpretation. We tested for differences between patients with primary and secondary PPPD. As no group differences were found, the data for both groups were collapsed. Data split by group is provided in the Supporting Information (Table S4).

Greenhouse–Geisser correction was applied for factors with more than two levels. Statistical significance was defined as *p* < 0.05. Post hoc comparisons were Bonferroni‐corrected for multiple testing. Spearman‐rho correlations were calculated to assess the relationships between egomotion ratings and symptom severity as measured by ALQ and NPQ. Comparisons were Bonferroni‐corrected for multiple testing, and insignificant results are shown in the Supporting Information.

## 3 Results

Analyzing the full‐factorial mixed ANOVA for factors: POSITION × VS × GVS × GROUP, we found a significant main effect of GROUP (*F*(1,45) = 25.16; *p* < 0.001; *η* ^2^ = 0.155), VS (*F*(1.29,57.90) = 25.27; *p* < 0.001; *η* ^2^ = 0.051), and GVS (*F*(1.54,69.22) = 333.22; *p* < 0.001; *η* ^2^ = 0.544) but no main effect of POSITION (*F*(1,45) = 0.159; *p* = 0.692; *η* ^2^ = 0.001). There was a significant interaction between VS × POSITION (*F*(1.66,74.96) = 6.28; *p* = 0.005; *η* ^2^ = 0.007), and VS × GVS (*F*(3.19,143.74) = 10.588; *p* < 0.001; *η* ^2^ = 0.014), while the remaining interactions did not reach significance (*p* always > 0.609).

**FIGURE 2:**
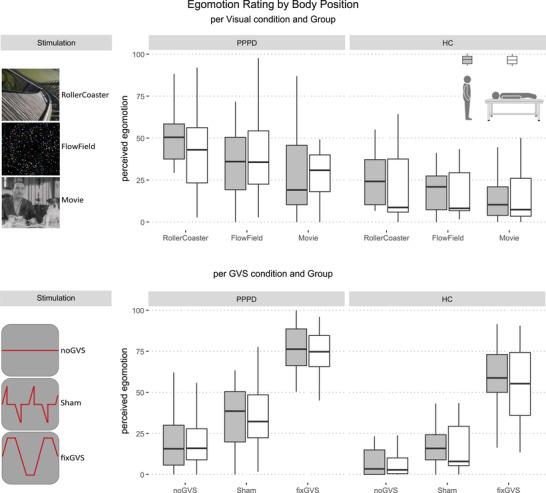
Boxplots depict perceived egomotion as a function of body position (standing = gray; supine = white), visual stimulation (VS) condition, galvanic vestibular stimulation (GVS) condition, and participant group. Upper row: Perceived egomotion by body position and Visual condition, separated by group: patients with persistent postural‐perceptual dizziness (PPPD) and healthy controls (HC). Lower row: Perceived egomotion by body position and GVS condition, separated by group (PPPD vs. HC). Boxes indicate the median and interquartile range; whiskers represent 1.5 times the interquartile range.

The mixed ANOVA for factors: POSITION × VS × GROUP (Figure 2, upper column) showed a significant main effect of GROUP (*F*(1,45) = 22.50; *p* < 0.001; *η* ^2^ = 0.181) and VS (*F*(1.44,64.62) = 25.60; *p* < 0.001; *η* ^2^ = 0.081) but no main effect of POSITION (*F*(1,45) = 0.23; *p* = 0.637; *η* ^2^ = 0.001). Furthermore, there was a significant interaction for VS × POSITION (*F*(1.62,72.75) = 3.84; *p* = 0.034; *η* ^2^ = 0.010). The remaining two‐ and three‐way interactions showed no significance. Post hoc comparisons of ratings in standing and supine body position per Visual condition and Group showed no significant differences in any of the standing versus supine ratings (*p* always > 0.05).

The mixed ANOVA for factors: POSITION x GVS x GROUP (Figure 2, lower column), showed a significant main effect of GROUP (*F*(1,45) = 21.47; *p* < 0.001; *η* ^2^ = 0.167) and GVS (*F*(1.54,69.10) = 308.85; *p* < 0.001; *η* ^2^ = 0.610) but no main effect of POSITION (*F*(1,45) = 0.004; *p* = 0.948; *η* ^2^ < 0.001) or a significant two‐ and three‐way interaction (*p* always > 0.05). Post hoc comparisons of ratings in standing and supine body position per GVS condition and Group showed no significant differences in any of the standing vs. supine ratings (*p* always > 0.05).

**FIGURE 3:**
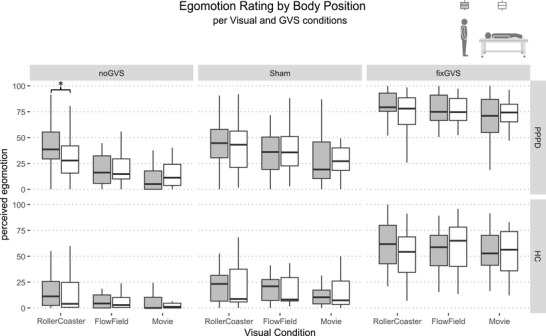
Boxplots depict perceived egomotion as a function of body position (standing = gray; supine = white), visual stimulation (VS) condition, galvanic vestibular stimulation (GVS) condition, and participant group. Upper column: patients with PPPD; Lower column: healthy controls (HC). Boxes indicate the median and interquartile range; whiskers represent 1.5 times the interquartile range. Asterisks indicate statistically significant effects for the body position (supine vs. standing) factor only, to enhance clarity. * *p* < 0.05.

The repeated‐measures ANOVA for factors POSITION × GVS × VS showed a significant main effect of VS (*F*(1.29,59.2) = 26.51; *p* < 0.001; *η* ^2^ = 0.046) and GVS (*F*(1.55,71.46) = 341.4; *p* < 0.001; *η* ^2^ = 0.504), but not POSITION (*F*(1,46) = 0.161; *p* = 0.691; *η* ^2^ < 0.001). Furthermore, there was significant interaction for VS × POSITION (*F*(1.64,75.65) = 6.95; *p* = 0.003; *η* ^2^ = 0.006) and VS × GVS (*F*(3.2,147.1) = 11.25; *p* < 0.001; *η* ^2^ = 0.013). Under the noGVS condition, analysis of the POSITION × VS × GROUP interaction (Figure 3) revealed significant main effects for GROUP (*F*(1,45) = 13.58; *p* = 0.001; *η* ^2^ = 0.130) and VS (*F*(1.35,60.84) = 38.81; *p* < 0.001; *η* ^2^ = 0.133) but not for POSITION (*F*(1,45) = 0.002; *p* = 0.965; *η* ^2^ < 0.001). There was a significant three‐way interaction between GROUP × VS × POSITION (*F*(1.6,72.01) = 4.54; *p* = 0.020; *η* ^2^ = 0.009). Furthermore, two significant two‐way interactions emerged: GROUP × VS (*F*(1.35,60.84) = 4.41; *p* = 0.029; *η* ^2^ = 0.017) and VS × POSITION (*F*(1.6,72.01) = 5.02; *p* = 0.014; *η* ^2^ = 0.010).

Post hoc comparisons indicated significantly larger egomotion ratings of PPPD patients in the standing, compared to the supine, position during rollercoaster stimulation (*p* = 0.029). No other pairwise comparisons reached significance. Under the sham and fixGVS conditions, analysis of the POSITION × VS × GROUP interaction revealed no significant three‐way interaction among the factors (*p* always > 0.05).

Correlation analysis between egomotion‐rating in standing and supine position with NPQ and ALQ scores revealed a significant correlation between the Niigata visual score and egomotion perception during visual stimulation in standing position (*ρ* = 0.441; *p* = 0.024).

## 4 Discussion

PPPD patients consistently reported stronger egomotion than healthy controls (HC), regardless of the stimulation condition (GVS, VS, or both). Both visual and vestibular inputs robustly influenced egomotion perception in standing and supine positions. This is crucial given that imaging studies in PPPD are performed in the supine position, without considering potential body position effects on egomotion perception.

With respect to the diagnostic criteria, egomotion perception did not differ between groups overall, even during complex visual motion (VS, Rollercoaster), challenging the view of a generally increased visual dependence of dizziness in PPPD. Visual dependence in PPPD does not necessarily reflect greater visual sensitivity, for example, visual motion perception thresholds were not lower but higher in PPPD patients, compared to HC, implying a poorer complex visual motion recognition (Storm et al. 2024).

Effects of visual motion stimulation seemed to be context‐ and body position‐dependent: Rollercoaster stimulation evoked stronger egomotion than Movie or FlowField stimulation in PPPD and HC (see Supporting Information Table S1), particularly in standing, suggesting a physiological response driven by increased postural demands. According to a cognitive–behavioral model of PPPD (Staab 2012), visually provocative stimuli elicit perceived postural threat and reflexive muscle stiffening and subsequently over‐reliance on vision. Accordingly, rollercoaster stimulation as the most postural destabilizing stimulus led to the highest egomotion ratings. This is in line with some context‐dependent abnormal visual sensitivity of PPPD patients in previous related studies: Patients showed larger postural sway, compared to HC, during visual exposure to rotating dots and scored higher on the Visual Vertigo Analogue Scale, compared to non‐PPPD dizzy patients and HC (De Vestel et al. 2022). Looking at functional brain activity, PPPD patients showed altered insular and visual‐cortex responses to vertical VR rollercoaster motion, compared to HC (Riccelli et al. 2017).

However, these studies did not record stimulus‐related egomotion perceptions, which is important to account for postural misperception as a proposed pathomechanism of dizziness in PPPD (Murillo et al. 2023). This postural misperception may result from an increased postural reliance on visual input and may be missed by general clinical scores of visual sensitivity (VVAS).

Notably, the context‐ and body‐position dependent effect disappeared when visual stimuli were combined with vestibular stimulation, underscoring the dominance of vestibular input in postural control. Both PPPD and HC showed similar response patterns in reaction to visual, vestibular and combined stimulation, arguing against impaired visual–vestibular interaction as a crucial pathomechanism in PPPD. However, it must be critically noted that the visual and vestibular stimulation were not matched in intensity, making it difficult to interpret a possible visual–vestibular interaction.

Unlike HC, patients showed a significant larger egomotion perception during rollercoaster VS in standing, compared to supine, position. This posture‐specific effect suggests abnormal weighting of visual cues for postural control in upright stance, in line with the diagnostic criteria. Differences in egomotion perception between body positions may come from conflicting sensory inputs. However, the latter were more likely to be expected in supine position as the viewing perspective in the lying position simulated an upward body position, while otoliths and somatosensory cues signaled supine. In contrast, PPPD patients showed stronger egomotion perception when standing, implying that the integration of strong visual motion cues becomes enhanced in the upright posture. This is specific to the complex rollercoaster VS. Future studies should examine how this altered perception influences postural control using posturography.

As outlined above, there are some potential limitations of our study (single‐center sample, non‐equivalent stimulus intensity on a perceptional level across modalities, using visual analogue scale as perceptual outcome measurement, differences in viewing geometry between standing and supine positions, cross‐sectional design) that should be taken into consideration for future comparisons.

Taken together, our findings point to a context‐dependent alteration of egomotion perception during upright stance. The correlation with the Niigata visual stimulation score underlines the clinical role of these findings (see Supporting Information Tables S2 and S3). As these differences disappeared with concomitant vestibular stimulation, it should be tested whether combined visual–vestibular rehabilitation reduces dizziness and abnormal egomotion in PPPD.

## Author Contributions

**Skadi Gerkensmeier**: writing – original draft, conceptualization, data curation, formal analysis, visualization, validation, methodology, writing – review and editing, investigation. **Hannah Keller**: methodology, data curation, writing – review and editing, investigation. **Pia Herborn**: data curation, methodology, writing – review and editing, investigation. **Renana Storm**: writing – review and editing, data curation, methodology, conceptualization, investigation. **Christoph Helmchen**: writing – original draft, conceptualization, investigation, project administration, supervision, resources, writing – review and editing, funding acquisition. **Andreas Sprenger**: conceptualization, investigation, validation, methodology, software, formal analysis, supervision, writing – review and editing.

## Funding

The study was supported by a research grant from the German Research Foundation to C.H. (HE 2689/6‐1).

## Ethics Statement

The study protocol was approved by the local Ethics Committee of the University of Lübeck (AZ 21–098). Written informed consent was obtained from all participants.

## Conflicts of Interest

The authors declare no conflicts of interest.

## Supporting information



Supporting Information: brb371268‐sup‐0001‐SuppMat.docx

## Data Availability

The data collected are not publicly available to preserve individuals’ privacy. The data are, however, available from the authors upon reasonable request.
