# Supplementary material for: Visual–Vestibular Modification of Egomotion Perception in Patients With Persistent Postural‐Perceptual Dizziness in Supine and Standing Positions
Source: Brain Behav. 2026 Feb 16;16(2):e71268. doi: 10.1002/brb3.71268 (PMC12910126; doi:10.1002/brb3.71268)
Supplement: Supplementary file 1 — Supporting Information: brb371268‐sup‐0001‐SuppMat.docx [file BRB3-16-e71268-s001.docx]

# Supplementary material

# Quantitative vestibular function testing:

Vestibular function was assessed using quantitative head impulse testing (qHIT), vestibular evoked myogenic potential testing, and quantification of the subjective visual vertical. The qHIT was assessed using the EyeSeeCam® HIT System (Autronics, Hamburg, Germany) at a sampling rate of 220 Hz. Quantitative HIT was delivered by passive head impulses (HIT) with rapid small amplitude (10–15°) horizontal head rotations (3000–4500°/s) while the participant was sitting on a chair fixating a red LED at a distance of 100 cm. The visual vertical was assessed with the head fixed on a chin rest by the subject’s adjustment of a bar to the perceived visual vertical without any spatial orientation clues in a dotted half-spherical dome, which is stationary or dynamic (moving visual background) around the line of sight. The normal range of SVV was defined as deviation of < 2.5°.

# Galvanic vestibular stimulation (GVS):

Galvanic stimulation was delivered using a low-frequency alternating current which passed between the two mastoid electrodes. The stimulation site was pre-treated with local anesthetics (Anesderm® lotion, Pierre Fabre Dermo-Kosmetik GmbH, Freiburg/Germany) 30min prior the experiment to minimize potential nociceptive stimulation. Individual vestibular thresholds were obtained by gradually decreasing and increasing the stimulus intensity in steps of 0.05mA, starting with an above-threshold current (1mA). We determined vestibular perceptional thresholds by GVS to make sure that we applied suprathreshold stimulations. All participants received GVS intensities above their individual threshold (mean GVS threshold for all patients: 0.27 mA). The procedure was repeated until a stable threshold was found. Depending on the condition, they received either no stimulation (noGVS), fixed-intensity galvanic vestibular stimulation (fixGVS: 1.3 mA with 100 ms linear onset and offset ramps, followed by a 300 ms stimulation plateau), or sham stimulation (sham: 1.3 mA with 100 ms linear onset but no stimulation plateau and sharp offset). The sham stimulus was designed to control for cutaneous sensations associated with electrode contact. Participants were informed that stimulation intensity could vary across trials, and both behavioral data and post-experiment debriefing indicated that the sham was perceived as a weaker form of stimulation. Moreover, previous posturography data from our group demonstrate that sham GVS does not affect postural sway in healthy controls (Helmchen et al., 2024). Stimulation duration was 20 seconds per trial.

# Visual Stimulation (VS):

For the RollerCoaster VS sections of publicly available point-of-view rollercoaster videos (see Video References) were trimmed to 20 second snippets. For the Movie VS sections of the movie “The Artist” (Hazanavicius, 2011) were trimmed to 20 second snippets. FlowField stimulation was programmed using Matlab (version R2022b, MathWorks, Natick/MA) and the Psychophysics Toolbox 3 extension (version 3.0.16, (Brainard, 1997)). In line with the paradigm used by (Ohlendorf et al., 2008), a central fixation point was continuously displayed throughout the stimulus presentation. The visual stimulus consisted of a dynamic cloud of colored dots originating from the center of the screen and radiating outward toward the periphery, where they gradually disappeared at the edges of the display. The motion trajectory followed a radial pattern, and the velocity of individual dots decreased continuously as a function of eccentricity.

# Body Position:

For measurements in the upright standing position, participants were instructed to stand upright with both feet placed parallel on the ground. Visual stimulation was presented on a height-adjustable computer screen positioned approximately 60 cm in front of the participant at eye level. In the supine condition, participants lay on their backs on a medical examination couch. Visual stimuli were presented on a computer screen, which participants viewed via an angled mirror positioned above their eyes.

# Statistical analysis:

All statistical analyses were conducted in R (version 4.4.3) using the RStudio interface (version 2024.12.1+563). The following packages were used (versions in parentheses): afex (1.4-1), emmeans (1.10.7), tidyverse (2.0.0), dplyr (1.1.4), ggplot2 (3.5.1), and ggpubr (0.6.0). All statistical analyses were initially conducted separately for patients with primary and secondary PPPD. However, as no significant differences in egomotion ratings were observed between the groups (p > .05 for all comparisons), data were subsequently pooled for all further analyses (post-hoc comparisons between groups in the main model are shown below: Suppl. Table 4). Assumption checks indicated that the ANOVA residuals were approximately normally distributed and that group variances were comparable (residuals-versus-fitted (QQ-)plots showed no pattern).

Spearman’s rho correlations were calculated to assess the relationships between egomotion ratings and symptom severity as measured by the Niigata Questionnaire (Yagi et al., 2019) and the Athens-Lübeck Questionnaire (ALQ)(Anagnostou et al., 2025). Specifically, we examined correlations between egomotion ratings in both standing and supine position during RollerCoaster VS and noGVS and the ALQ subscale ALQvis and the Niigata visual stimulation score. Furthermore, we calculated the correlation during upright standing without any stimulation and the ALQstand and Niigata upright posture score.

Power estimation

Statistical power was estimated using simulation-based methods implemented in the R package *simr* (Green & MacLeod, 2016). Power simulations were based on a linear mixed-effects model, including the fixed effects GROUP, GVS, VS, and POSITION and their interactions, with subject included as a random intercept.

Model parameters were specified by simulating data under explicit assumptions about the main effects of GROUP, GVS, and VS, as well as the two-way interactions GROUP × GVS and GROUP × VS, derived from prior empirical findings. No assumptions were made regarding the main effect of POSITION or any higher-order interactions, which were therefore set to zero in the data-generating model. Residual and random-intercept variances were chosen to reflect variability observed in previous experiments.

For each simulated dataset, a likelihood ratio test was performed comparing the full and reduced models to determine whether the interactions contributed significantly to the model fit, and power was estimated as the proportion of simulations in which the test was significant at α = 0.05

Simulations were conducted assuming a sample size of 20 subjects per group (N = 40 total), with each subject contributing repeated measurements across all experimental conditions. Based on 200 simulations, the estimated power to detect the interaction effects was 84.5% (95% CI: 78.7%–89.2%) at an alpha level of α = 0.05.

# Additional Results

**Suppl. Table 1.**: Post hoc pairwise comparisons: Visual stimulation (noGVS) within Group

| Group | Contrast | Estimate | SE | df | t-ratio | p-value | Cohen’s d |  |
| --- | --- | --- | --- | --- | --- | --- | --- | --- |
| PPPD | RollerCoaster - FlowField | 15.838 | 2.918 | 45 | 5.427 | **<0.001** | 0.809 |  |
| PPPD | RollerCoaster - Movie | 22.215 | 3.102 | 45 | 7.161 | **<0.001** | 1.068 |  |
| PPPD | FlowField - Movie | 6.377 | 1.457 | 45 | 4.378 | **<0.001** | 0.653 |  |
| HC | RollerCoaster - FlowField | 8.486 | 3.247 | 45 | 2.613 | **0.024** | 0.390 |  |
| HC | RollerCoaster - Movie | 10.830 | 3.452 | 45 | 3.138 | **0.009** | 0.468 |  |
| HC | FlowField - Movie | 2.344 | 1.621 | 45 | 1.446 | 0.155 | 0.216 |  |

**Suppl. Table 2**.: Spearman's rho correlation between ALQ subscores and Rating (PPPD)

| Correlation | r | p |
| --- | --- | --- |
| **ALQstand x Standing (noGVS/NoVisual)** | 0.041 | 0.841 |
| **ALQvis x Standing (noGVS/Rollercoaster)** | 0.298 | 0.139 |
| **ALQvis x Supine (noGVS/Rollercoaster)** | -0.018 | 0.932 |

**Suppl. Table 3.**: Spearman's rho correlation between Niigata subscores and Rating (PPPD)

| Correlation | r | p |
| --- | --- | --- |
| **Niigata upright posture x Standing (noGVS/NoVisual)** | 0.211 | 0.302 |
| **Niigata visual stimulation x Standing (noGVS/Rollercoaster)** | 0.441 | **0.024** |
| **Niigata visual stimulation x Supine (noGVS/Rollercoaster)** | 0.163 | 0.427 |

**Suppl. Table 4.:** Post-Hoc comparison of Groups for the VSxGSxPositionxGroup ANOVA

| Contrast | Estimate | SE | df | t-ratio | p-value | Cohen’s d |
| --- | --- | --- | --- | --- | --- | --- |
| PPPD primary - PPPD secondary | -2.219 | 4.567 | 44 | -0.486 | 0.629 | -0.073 |
| PPPD primary - HC | 15.677 | 3.759 | 44 | 4.170 | **< 0.001** | 0.629 |
| PPPD secondary - HC | 17.896 | 4.353 | 44 | 4.112 | **< 0.001** | 0.620 |

**Suppl. Table 5.:** Median and IQR per Group × GVS × Visual × Position

| Group | GVS | Visual | Position | Median | IQR |
| --- | --- | --- | --- | --- | --- |
| PPPD | noGVS | RollerCoaster | standing | 38.55 | 26.01 |
| PPPD | noGVS | RollerCoaster | supine | 27.74 | 26.33 |
| PPPD | noGVS | FlowField | standing | 16.10 | 26.61 |
| PPPD | noGVS | FlowField | supine | 14.69 | 19.41 |
| PPPD | noGVS | Movie | standing | 5.00 | 17.72 |
| PPPD | noGVS | Movie | supine | 11.18 | 20.26 |
| PPPD | Sham | RollerCoaster | standing | 44.56 | 27.48 |
| PPPD | Sham | RollerCoaster | supine | 43.02 | 34.98 |
| PPPD | Sham | FlowField | standing | 36.00 | 31.24 |
| PPPD | Sham | FlowField | supine | 35.63 | 28.30 |
| PPPD | Sham | Movie | standing | 19.11 | 35.40 |
| PPPD | Sham | Movie | supine | 27.16 | 21.83 |
| PPPD | fixGVS | RollerCoaster | standing | 79.27 | 17.51 |
| PPPD | fixGVS | RollerCoaster | supine | 77.98 | 25.91 |
| PPPD | fixGVS | FlowField | standing | 74.84 | 24.49 |
| PPPD | fixGVS | FlowField | supine | 74.55 | 21.29 |
| PPPD | fixGVS | Movie | standing | 70.98 | 32.04 |
| PPPD | fixGVS | Movie | supine | 74.21 | 16.99 |
| HC | noGVS | RollerCoaster | standing | 11.24 | 23.14 |
| HC | noGVS | RollerCoaster | supine | 3.99 | 24.15 |
| HC | noGVS | FlowField | standing | 4.30 | 12.63 |
| HC | noGVS | FlowField | supine | 2.82 | 9.66 |
| HC | noGVS | Movie | standing | 0.00 | 10.32 |
| HC | noGVS | Movie | supine | 1.12 | 4.63 |
| HC | Sham | RollerCoaster | standing | 23.27 | 24.99 |
| HC | Sham | RollerCoaster | supine | 8.68 | 31.74 |
| HC | Sham | FlowField | standing | 20.96 | 20.13 |
| HC | Sham | FlowField | supine | 8.15 | 22.58 |
| HC | Sham | Movie | standing | 10.32 | 13.30 |
| HC | Sham | Movie | supine | 7.40 | 22.58 |
| HC | fixGVS | RollerCoaster | standing | 61.76 | 37.25 |
| HC | fixGVS | RollerCoaster | supine | 54.23 | 34.28 |
| HC | fixGVS | FlowField | standing | 58.76 | 29.42 |
| HC | fixGVS | FlowField | supine | 65.00 | 37.99 |
| HC | fixGVS | Movie | standing | 52.77 | 28.96 |
| HC | fixGVS | Movie | supine | 56.35 | 37.90 |

# References

Anagnostou, E., Armenis, G., Zachou, A., Storm, R., Sprenger, A., & Helmchen, C. (2025). The Athens-Lubeck Questionnaire: a tool to discriminate between subtypes of persistent postural perceptual dizziness. *Front Neurol*, *16*, 1550469. <https://doi.org/10.3389/fneur.2025.1550469>

Brainard, D. H. (1997). The Psychophysics Toolbox. *Spat Vis*, *10*(4), 433-436. <https://www.ncbi.nlm.nih.gov/pubmed/9176952>

Green, P., & MacLeod, C. J. (2016). SIMR: An R package for power analysis of generalized linear mixed models by simulation. *Methods in Ecology and Evolution*, *7*(4), 493-498.

Helmchen, C., Blum, S. K., Storm, R., Krause, J., & Sprenger, A. (2024). Postural motion perception during vestibular stimulation depends on the motion perception threshold in persistent postural-perceptual dizziness. *J Neurol*, *271*(8), 4909-4924. <https://doi.org/10.1007/s00415-024-12415-z>

Ohlendorf, S., Sprenger, A., Speck, O., Haller, S., & Kimmig, H. (2008). Optic flow stimuli in and near the visual field centre: a group FMRI study of motion sensitive regions. *PLoS ONE*, *3*(12), e4043. <https://doi.org/10.1371/journal.pone.0004043>

Yagi, C., Morita, Y., Kitazawa, M., Nonomura, Y., Yamagishi, T., Ohshima, S., Izumi, S., Takahashi, K., & Horii, A. (2019). A Validated Questionnaire to Assess the Severity of Persistent Postural-Perceptual Dizziness (PPPD): The Niigata PPPD Questionnaire (NPQ). *Otol Neurotol*, *40*(7), e747-e752. <https://doi.org/10.1097/MAO.0000000000002325>

# Video References

Hazanavicius, M. (Director). (2011). The Artist [Film]. La Petite Reine**;** Studio 37**;** La Classe Américaine**;** JD Prod**;** France 3 Cinéma**;** Jouror Productions**;** uFilm**.**

4K AWESOME Twister Roller Coaster Front Seat POV Knoebels Amusement Park. Available (as of May, 2025): <https://www.youtube.com/watch?v=oAJLKDMihnU>

Tremors Front Row POV | Silverwood Theme Park 2022. Available (as of May, 2025): <https://www.youtube.com/watch?v=zgU8bQDpn-I>

Nitro front seat on-ride 4K POV @60fps Six Flags Great Adventure**.** Available (as of May, 2025)**:** <https://www.youtube.com/watch?v=ZmMLSUbRuoM>
